# Exploring the Acceptance of Just-in-Time Adaptive Lifestyle Support for People With Type 2 Diabetes: Qualitative Acceptability Study

**DOI:** 10.2196/65026

**Published:** 2025-02-19

**Authors:** Eclaire A G Hietbrink, Anouk Middelweerd, Wendy d’Hollosy, Laura K Schrijver, Gozewijn D Laverman, Miriam M R Vollenbroek-Hutten

**Affiliations:** 1 Department of Biomedical Signals and Systems University of Twente Enschede The Netherlands; 2 Department of Internal Medicine/Nephrology Ziekenhuisgroep Twente Almelo The Netherlands; 3 Medisch Spectrum Twente Enschede The Netherlands

**Keywords:** eHealth, just-in-time adaptive intervention, ecological momentary assessment, type 2 diabetes, behavior change, physical activity, nutrition, acceptability, formative evaluation, mobile phone

## Abstract

**Background:**

The management of type 2 diabetes (T2D) requires individuals to adopt and maintain a healthy lifestyle. Personalized eHealth interventions can help individuals change their lifestyle behavior. Specifically, just-in-time adaptive interventions (JITAIs) offer a promising approach to provide tailored support to encourage healthy behaviors. Low-effort self-reporting via ecological momentary assessment (EMA) can provide insights into individuals’ experiences and environmental factors and thus improve JITAI support, particularly for conditions that cannot be measured by sensors. We developed an EMA-driven JITAI to offer tailored support for various personal and environmental factors influencing healthy behavior in individuals with T2D.

**Objective:**

This study aimed to assess the acceptability of EMA-driven, just-in-time adaptive lifestyle support in individuals with T2D.

**Methods:**

In total, 8 individuals with T2D used the JITAI for 2 weeks. Participants completed daily EMAs about their activity, location, mood, overall condition, weather, and cravings and received tailored support via SMS text messaging. The acceptability of the JITAI was assessed through telephone-conducted, semistructured interviews. Interview topics included the acceptability of the EMA content and prompts, the intervention options, and the overall use of the JITAI. Data were analyzed using a hybrid approach of thematic analysis.

**Results:**

Participants with a mean age of 70.5 (SD 9) years, BMI of 32.1 (SD 5.3) kg/m², and T2D duration of 15.6 (SD 7.7) years had high self-efficacy scores in physical activity (ie, 32) and nutrition (ie, 29) and were mainly initiating or maintaining behavior changes. The identified themes were related to the intervention design, decision points, tailoring variables, intervention options, and mechanisms underlying adherence and retention. Participants provided positive feedback on several aspects of the JITAI, such as the motivating and enjoyable messages that appeared well tailored to some individuals. However, there were notable differences in individual experiences with the JITAI, particularly regarding intervention intensity and the perceived personalization of the EMA and messages. The EMA was perceived as easy to use and low in burden, but participants felt it provided too much of a snapshot and too little context, reducing the perceived tailoring of the intervention options. Challenges with the timing and frequency of prompts and the relevance of some tailoring variables were also observed. While some participants found the support relevant and motivating, others were less inclined to follow the advice. Participants expressed the need for even more personalized support tailored to their specific characteristics and circumstances.

**Conclusions:**

This study showed that an EMA-driven JITAI can provide motivating and tailored support, but more personalization is needed to ensure that the lifestyle support more closely fits each individual’s unique needs. Key areas for improvement include developing more individually tailored interventions, improving assessment methods to balance active and passive data collection, and integrating JITAIs within comprehensive lifestyle interventions.

## Introduction

### Background

A healthy lifestyle has a pivotal role in effective management of type 2 diabetes (T2D) [1,2]. This approach includes addressing important contributors to T2D, such as insufficient physical activity, unhealthy dietary habits, inadequate sleep, and elevated stress levels. Substantial scientific evidence supports the substantial health benefits of lifestyle interventions [3-8], including studies indicating that lifestyle modifications can potentially reverse or achieve remission in T2D. In addition, a 10-year study demonstrated that lifestyle interventions could result in economic benefits in the form of reduced health care costs [9].

However, individuals with T2D often struggle to adhere to lifestyle guidelines [10-13]. One explanation is that social and environmental changes, such as the prevalence of sedentary jobs and the widespread availability of highly processed foods, have made it increasingly challenging for individuals to adopt and maintain a healthy lifestyle [14]. Moreover, managing a chronic condition poses additional challenges, including the time and effort required to manage symptoms, adhere to treatment plans, and cope with physical and psychosocial consequences [15,16]. Individuals need knowledge and self-management skills to implement sustainable lifestyle changes [2]. Due to variations in personal, environmental, and disease-related factors, self-management skills can differ substantially from person to person [17]. Therefore, a personalized approach is essential to guide each individual toward a healthier lifestyle, providing tailored support that meets their unique needs.

eHealth, which involves using technology to promote health, well-being, and health care [18], holds considerable potential in delivering personalized lifestyle support. Research has demonstrated that eHealth interventions can be both acceptable and effective for improving physical activity, diet, and health outcomes [19-23]. A promising approach to provide personalized support is just-in-time adaptive interventions (JITAIs). JITAIs are designed to dynamically provide the right support at the right time [24]. A key element of JITAIs is the need for insights into behavior and context to adapt the support accordingly. Today, wearables, smartphone sensors, and remote monitoring devices, such as activity trackers, GPS, and smartphone calendars, can be used for real-time monitoring and to inform intervention decisions [24,25]. In addition, low-effort self-reporting via ecological momentary assessment (EMA) can provide further insights into an individual’s situation and emotional state, which may improve the support offered by JITAIs [26]. EMA involves repeated sampling of an individual’s behaviors, cognitions, emotions, and environmental factors in their natural environment through short questionnaires that are prompted multiple times within a certain time frame [27]. EMAs allow for adapting support to individual needs, particularly for conditions that cannot be measured by sensors. Unlike typical eHealth interventions, JITAIs can provide near–real-time and contextually relevant support, tailored to an individual’s needs and preferences [24].

JITAIs have been studied to determine their added value in supporting several lifestyle behaviors, such as physical activity, diet, and substance use. Studies have examined their acceptability; feasibility; and, to a lesser extent, effectiveness [28-32]. Overall, JITAIs appear to be acceptable, although some feasibility challenges were identified, including issues with smartphone battery life, sensor reliability, the punctuality of JITAI messages, and the incomplete automation of JITAI delivery [28,31]. People were more likely to follow JITAI messages and to find them more relevant than randomly selected messages [33,34]. Evidence on the effectiveness of JITAIs for behavior change is mixed, and there is insufficient data to draw well-founded conclusions [28,29,32]. JITAIs that use algorithms to tailor interventions based on past behavior and current needs, along with guidance from a human, are more effective [30].

### Objectives

Our research team previously developed the E-Supporter, an evidence-based and tailored digital coach for people with chronic conditions, primarily T2D [35]. A previous feasibility study on the E-Supporter revealed that individuals with T2D need more tailored support to better align with their personal circumstances [36]. This finding highlights the need for interventions that can adapt to individual needs. JITAIs offer the opportunity to provide personalized, contextually relevant support when it is needed. In many JITAIs, tailoring is often based on objective parameters related to healthy lifestyles, such as wearable data [37-39]. However, these eHealth interventions do not frequently address the more nuanced aspects of individuals’ experiences, such as disease-related challenges or environmental factors [28]. While a few JITAIs assess these conditions via EMA [26,32], none currently tailor support to the unique characteristics of individuals with T2D. Therefore, we developed an EMA-driven JITAI designed to offer tailored assistance for several personal and environmental factors that influence healthy behavior. Given its novelty, assessing acceptability is crucial for refinements and future development [18,40]. Hence, we conducted an acceptability assessment for the EMA-driven JITAI designed to support a healthy lifestyle for individuals with T2D.

## Methods

### Study Design

We performed a qualitative study to assess the acceptability of the JITAI over 2 weeks among individuals with T2D. The JITAI was designed to support physical activity and healthy nutrition in individuals with T2D. The study was conducted during a national lockdown due to COVID-19, requiring all contact with participants to take place remotely.

### Intervention Description

#### Overview

The foundation of the developed JITAI is rooted in the E-Supporter 1.0 [35], designed to promote physical activity and a healthy diet among individuals with chronic diseases, initially focusing on T2D. The E-Supporter 1.0 was participatory developed with input from both patients with T2D and health care professionals. It is tailored to several variables, including behavioral objectives, the phase of behavior change, type of chronic disease, time of day, and goal achievement. The behavior change strategies are based on the *Health Action Process Approach* [41] and theories to support behavior maintenance [42,43]. An in-depth description of the E-Supporter 1.0 can be found elsewhere [35].

**Figure 1:**
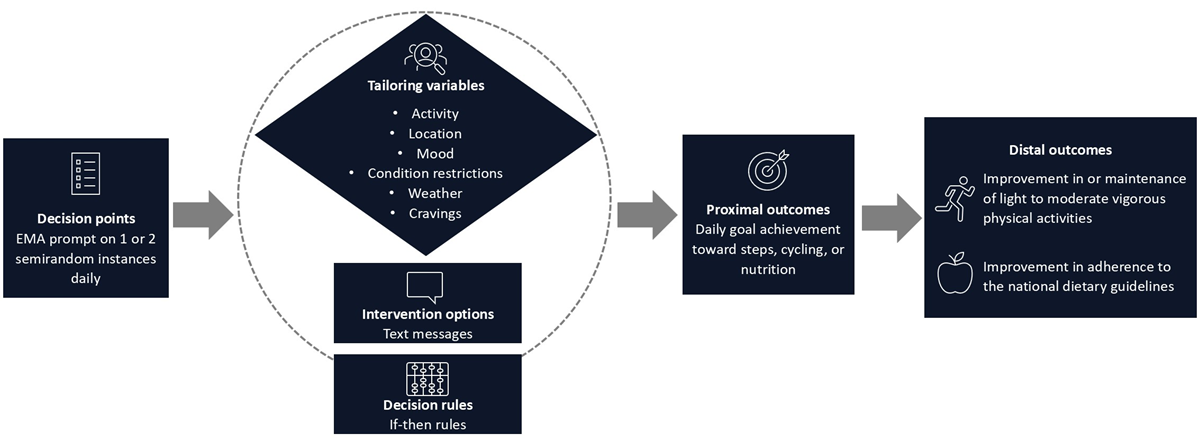
Overview of the intervention components based on the conceptual model of just-in-time adaptive interventions. EMA: ecological momentary assessment.

We used a systematic approach to design the JITAI according to the principles outlined in the framework by Nahum-Shani et al [24]. This process involved developing the 6 components of a JITAI: distal outcome, proximal outcome, tailoring variables, intervention options, decision rules, and decision points (Figure 1 [24]). The development process involved iterative feedback on all components from a group of multidisciplinary experts, including a health scientist, sports scientist, computer scientist, and diabetologist.

#### Distal Outcome

The distal outcome represents *the ultimate long-term goal* of the intervention, indicating the desired outcome that is expected to be achieved [24]. The distal outcome regarding the physical activity module is improvement in, or maintenance of, light to moderate vigorous physical activities in people with T2D. Regarding the nutritional module, the distal outcome is improvement in adherence to the national dietary guidelines [44]. A substantiation for the choice of the distal outcomes can be found in the study by Hietbrink et al [35].

#### Proximal Outcome

The proximal outcome represents *the short-term goal* that the intervention aims to achieve and indicates the progress to the distal outcome [24]. For this JITAI, we opted for a proximal outcome centered on actual behavior. The E-Supporter 1.0 allows users to set specific goals for steps, cycling, or nutrition, contributing to the attainment of distal outcomes [35]. Consequently, we focused on *daily goal achievement* as our proximal outcome, which is considered a meaningful indicator of short-term behavioral progress toward the distal outcome [24].

#### Tailoring Variables

Tailoring variables refer to individual-specific information used for personalization, determining when and how to intervene [24]. To identify tailoring variables, we conducted a survey assessing facilitators and barriers for a healthy lifestyle among individuals with a lifestyle-related chronic condition (L K Schrijver, MSc, unpublished data, March 2020). In the domain of physical activity, important dynamic factors included weather, mood, daily activities, fatigue, disease symptoms, pain, and social support. Regarding dietary choices, mood, location (eg, eating out in a restaurant), social occasions, and the presence of unhealthy foods emerged as dynamic factors. The research group clustered these factors into the tailoring variables for the JITAI. The following 6 tailoring variables were identified: activity, location, mood, physical or conditional restrictions, weather, and cravings. In this study, we used EMA as an active assessment method (ie, input from the user is required) to evaluate all tailoring variables. Each variable was addressed through a specific EMA question. The research team designed, evaluated, and refined the EMA questions and corresponding response options. Table 1 presents an overview of the tailored EMA questions designed to assess the identified variables.

**Table 1:** Ecological momentary assessment questions and corresponding response options designed to assess the tailoring variables during the study period.

Tailoring variable	Question	Response options	Coaching domain (ie, physical activity or nutrition)
Activity	What are you currently doing?	At work or studying Leisure activity Social occasion Traveling Eating (ie, other than a social occasion) Being physically active Otherwise	Both
Location	What is your current location?	At home At someone else’s house At work In a vehicle In a restaurant or café or on a terrace At a sports club or other sports location Outside, not otherwise specified Otherwise	Both
Mood	How are you feeling at the moment?	I feel positive emotions (eg, happy, cheerful, loved, confident, or relaxed) I feel negative emotions (eg, sad, angry, stressed, ashamed, or anxious) I feel neutral (neither positive nor negative)	Both
Restrictions in physical or mental condition	Are you currently experiencing restrictions in your overall condition?	I experience no restrictions I experience discomfort due to my physical limitations I experience pain complaints I experience diabetes-specific symptoms (eg, hyperglycemia or hypoglycemia) I experience fatigue Otherwise	Physical activity
Weather	How do you feel about the weather for doing physical activity outdoors?	The weather is nice to be physically active outside The weather is not suitable (ie, too hot, cold, or wet) to be physically active outside	Physical activity
Cravings	Are you currently craving food?	No, I do not feel like eating Yes, I feel like eating healthy Yes, I feel like eating unhealthy Yes, but I do not crave a specific food	Nutrition

#### Intervention Options

Intervention options represent the diverse behavior change actions that can be taken in response to decision points [24]. The content of the intervention options was rooted in the theoretical framework of the E-Supporter 1.0 [35], structured around 3 behavioral change phases: initiation, action, and maintenance. For each phase, relevant determinants of behavior were identified, and corresponding behavior change techniques (BCTs) were integrated to influence behavior effectively. BCTs are active components included in interventions designed to change behavior through various mechanisms [45].

The JITAI contained a set of 215 short SMS text messages, each tailored to address the tailoring variables (Table 2). These messages aimed to build skills, motivate, prompt reflection, and provide encouragement. Input to develop the intervention options was gathered from the following sources: the literature, the Dutch Diabetes Fund, the Dutch Nutrition Center, and the Dutch guidelines for physical activity and healthy diet [44,46]. The determinants and BCTs from the E-Supporter 1.0 were used as basis to develop the JITAI intervention options [35]. In addition, new determinants and BCTs were added to align with the selected tailoring variables, such as activity and location, which use environmental cues and context to prompt and guide individuals in various settings (eg, work, home, or during specific activities) [47]. For a comprehensive overview of determinants and BCTs, please refer to Multimedia Appendix 1.

**Table 2:** Examples of intervention options provided during the study, featuring tailored text messages customized for each tailoring variable.

Tailoring variable	Answer option	Example intervention option
Activity	At work or studying	“Do you have a sedentary job? Scheduling a daily walk during your break can help. Maybe your colleagues would like to join you. It is not only good for physical activity but also a fun way to spend time together!”
Location	In a restaurant or café or on a terrace	“Hi! Eating out at a restaurant? Try to choose the healthier option on the menu. Here are some examples of fairly healthy main courses: lean meat (eg, chicken or turkey fillet) or fatty fish (eg, salmon), served with vegetables, potatoes, or whole grain rice.”
Weather	The weather is nice to be physically active outside	“Hello *[name]*, with this beautiful weather, everything around you looks even nicer. Take a walk around the neighborhood and enjoy it!”
Mood	I feel negative emotions (think sad, angry, stressed, angry, ashamed, or anxious)	“Hello *[name]*, I am sorry you are not feeling great. Even when you are feeling down, a walk might help. Give it a try, it could do you some good!”
Physical restrictions	I experience fatigue	“Hi, Feeling tired? You can break up your daily physical activity into shorter sessions. That way, you will have time to rest in between.”
Food cravings	Yes, I feel like eating unhealthy food	“If the temptation to snack is strong, doing something else can help, like going for a walk or talking to someone. A little distraction can make the craving fade!”

#### Decision Rules

The decision rules serve as the link between tailoring variables and intervention options, enabling personalized adaptation of the intervention based on when, for whom, and which intervention option to offer [24]. All intervention options were systematically coded according to a behavioral goal, phase of behavior change, determinants of behavior, BCTs, tailoring variable from the EMA, and time of day. In our intervention, we used rule-based decisions with if-then statements—if certain conditions were met, then a specific intervention option was provided. A fitting intervention option was randomly selected. In instances where none of the assessed tailoring variables influenced health behavior or the EMA was not filled out, a text message was sent from the message set derived from the previously developed E-Supporter 1.0.

#### Decision Points

A decision point refers to a specific moment in time when the delivery of interventions to the user is determined [24]. In this JITAI, intervention decisions were triggered by each EMA prompt. EMAs were initiated through time-based sampling, occurring at 1 or 2 semirandom instances daily—once in the morning and again in the afternoon or evening.

### Participants and Recruitment

We aimed to recruit 8 to 10 individuals diagnosed with T2D for this study, as this group size typically yields valuable insights into the acceptability of technologies [48]. To be eligible for participation, individuals needed to have a diagnosis of T2D, be aged ≥18 years, own a mobile phone, have the ability to understand Dutch, and possess the capacity to understand the informed consent procedure. Participants were recruited from the observational Diabetes and Lifestyle Cohort Twente study, a longitudinal study to obtain lifestyle, glucose, and disease-related parameters in patients with T2D [49], from Ziekenhuisgroep Twente (ZGT), a regional hospital in the east of the Netherlands. ZGT has a diabetes outpatient clinic where internists, nurse practitioners, and diabetes nurses provide daily care for a large population of patients with T2D. This hospital is also actively engaged in extensive scientific research related to diabetes. Individuals from the Diabetes and Lifestyle Cohort Twente cohort who had previously expressed willingness to participate in additional scientific research were contacted by telephone by the researcher (EAGH) to determine their interest in participating in this study. Detailed study information was provided orally, and interested individuals were sent the participant information sheet via email. After a week, potential participants were contacted again by telephone to inquire about their decision to participate. Those who chose to participate provided their informed consent by signing a digital or paper consent form before study enrollment. Although participants were recruited from ZGT hospital, participants used the JITAI in their home situation.

### Procedures

The researcher introduced herself and the aims of the study before the start of the intervention. Participants were instructed to use the intervention for a period of 2 weeks, with the option to receive coaching for physical activity, healthy nutrition, or both lifestyle domains. Before starting the intervention, participants completed a web-based survey, providing questionnaires related to self-efficacy and phase of behavior change (refer to the Data Collection and Analysis section). The information on participants’ self-efficacy and phase of behavior change was used to identify the most suitable self-efficacy messages and phase of behavior change messages for delivering intervention options (eg, people in the action phase received fewer messages related to behavior initiation and more messages regarding the action and maintenance phase) [35].

Throughout the intervention, participants were prompted to fill in the EMAs at 1 or 2 semirandom time intervals each day—one in the morning and another in the afternoon or evening [26]. At the prompt time, participants received a text message containing a link to access the EMA questionnaire. Intervention options were delivered via texting, with a maximum of 2 messages per day.

Following the 2-week intervention period, semistructured interviews were conducted via telephone and audio recorded by EAGH, a female PhD candidate in health sciences, to gather in-depth insights and feedback from participants regarding their experiences with the intervention. The interviewer followed a preestablished interview guide, had prior experience in conducting interviews, and had no prior relationship with the study participants. Each interview lasted between 20 and 40 minutes.

### Data Collection and Analysis

#### Acceptability

After the 2-week intervention period, participants engaged in a semistructured interview to elicit their thoughts, experiences, and suggestions for improvement. We examined the comprehensiveness and ease of use regarding the use of the JITAI [18]. Furthermore, the focus was primarily on satisfaction with the experience and the perceived usefulness of the intervention [40]. The interview schedule (Multimedia Appendix 2) covered three core parts: (1) acceptability of the EMA content and prompts, (2) acceptability of the intervention options (ie, the text messages), and (3) use of the JITAI (ie, the combinations of EMA and the messages).

The interview questions regarding the EMA questionnaire section were based on recommendations to assess the acceptability of EMAs [50], ensuring questions regarding item relevance, comprehensiveness, and instrument comprehensibility. In addition, participants’ perceptions of EMA burden in terms of length, intensity, and motivation for completion were addressed. To evaluate the intervention options and the use of the JITAI, the interview schedule was based on the *Unified Theory of Acceptance and Use of Technology 2* framework [51]. Questions aligned with the *Unified Theory of Acceptance and Use of Technology 2* determinants, such as performance expectancy, effort expectancy, use behavior, and behavioral intention.

The interviews were analyzed based on a hybrid approach of thematic analysis, as outlined by Bingham [52] using ATLAS.ti 23 (Lumivero, LLC). This method combines both inductive and deductive strategies, enabling the development of new insights from the data while grounding the findings in an existing theoretical framework. The inductive phase began with reviewing the transcripts to familiarize with participants’ responses, followed by an open inductive coding approach. Each text segment offering a notable perspective on the research questions received a descriptive open code. EAGH coded all transcripts, while AM independently coded 25% (2/8) and cross-checked the remainder. Open codes were iteratively refined, and discrepancies between EAGH and AM were resolved through discussions until consensus was achieved. The process of axial coding followed, involving clustering and combining open codes to identify patterns within and across interviews. Themes were developed by incorporating axial codes into theme statements that captured the observed data patterns. In the deductive phase, the *conceptual model of JITAI components* proposed by Nahum-Shani et al [24] was used to refine and classify the theme statements based on JITAI’s main intervention components. Initially, themes were broadly assigned to the JITAI component where the definitions showed the greatest overlap. This classification was iteratively revised, with themes being reassigned until all themes were grouped under the most appropriate intervention component within the model. Throughout these phases, the research team continually discussed and refined the findings until consensus was reached.

#### Self-Efficacy Level

Self-efficacy was measured using the Exercise Self-Efficacy Scale (ESES) [53], consisting of 10 items that assess self-confidence in performing physical activity. In addition, a modified version of the ESES was used to determine self-efficacy related to a healthy diet [54]. The assessment used a 4-point Likert scale, ranging from *1 (ie, not at all true)* to *4 (ie, always true)*, yielding sum scores between 10 and 40, with higher scores indicating higher self-efficacy levels. Self-efficacy scores were treated as a continuous variable, obtained by summing the scores from the 10 items of the ESES questionnaire. Mean (SD) was used to present self-efficacy scores.

#### Phase of Behavior Change

To assess the phase of behavior change, single-question self-assessment stages of change scale [55] was used. This scale uses a single question to inquire about an individual’s engagement in healthy behavior, aligning with the 5 phases of behavior change described in the transtheoretical model [56]. The phase of behavior change was categorized as an ordinal variable, ranging from 1 to 5, representing the following phases: (1) maintenance, (2) action, (3) preparation, (4) contemplation, and (5) precontemplation. These 5 answer options were aligned with the 3 behavior change phases targeted in the E-Supporter 1.0 intervention to allow for tailoring of intervention content. Participants who selected answer options 3, 4, or 5 were in the initiation phase, participants who chose answer option 2 were classified in the action phase, and participants who selected answer option 1 were categorized in the maintenance phase.

#### Demographics

Information on age (years), sex (ie, male, female, or other), BMI (kg/m^2^), duration of T2D diagnosis (years), medication use (ie, none, oral, or insulin), and diabetes-related complications (ie, none, retinopathy, nephropathy, or neuropathy) was obtained from the hospital’s electronic patient record. Having a job (ie, yes or no) and highest level of education (ie, no formal education, elementary education, lower secondary education, upper secondary education, higher vocational education, preuniversity education, university education, or postgraduate education) were questioned. Demographic characteristics were reported as mean (SD) for continuous variables or frequency (%) for categorical variables.

### Ethical Considerations

The study protocol was reviewed and approved by the Medical Ethics Research Committee Twente, Enschede, the Netherlands (K20-05). In addition, the local advisory committee of practical feasibility in ZGT hospital approved to conduct this study (ZGT17-39). We obtained informed consent from all study participants, either digitally or on paper. Participants did not receive financial compensation for their participation due to the short duration of the study. Audio recordings of the interviews were deleted after transcription. All participant-identifiable data were removed or pseudonymized. The data are stored on a secure, encrypted server at the University of Twente, accessible only to the researchers.

## Results

### Participant Characteristics

In total, 12 individuals were invited to participate in the study. Three people declined participation due to personal circumstances or perceiving the study burden as too high. One participant initially agreed but withdrew prematurely due to family circumstances. The characteristics of the 8 participants who completed the study are presented in Table 3. The participants had a mean age of 70.5 (SD 9.0) years, a mean BMI of 32.1 (SD 5.3) kg/m^2^, and a mean T2D diagnosis of 15.6 (SD 7.7) years. Microvascular complications included neuropathy in 50% (4/8) of the participants and nephropathy in 25% (2/8) of the participants. In addition, of the 8 participants, 5 (63%) were using insulin and 6 (75%) remained active in work or volunteer activities. The most common educational level was higher vocational education (4/8, 50%).

A total of 6 participants opted for coaching on both physical activity and nutrition, while 1 participant preferred coaching exclusively on physical activity and another participant preferred coaching solely on nutrition. The results indicate that participants reported higher self-efficacy levels in the domain of nutrition compared to physical activity, with mean scores of 32 (SD 4) and 29 (SD 7), respectively (Table 4). Regarding the phase of behavior change, a large proportion of participants were in the maintenance phase for both physical activity (3/7, 43%) and nutrition (5/7, 71%).

**Table 3:** Participant characteristics (n=8).

Characteristic		Participants
Age (y), mean (SD)		70.5 (9)
**Sex, n (%)**		
	Male	6 (75)
	Female	2 (25)
BMI (kg/m^2^), mean (SD)		32.1 (5.3)
Type 2 diabetes duration (y), mean (SD)		15.6 (7.7)
**Complications, n (%)**		
	Retinopathy	0 (0)
	Neuropathy	4 (50)
	Nephropathy	2 (25)
Insulin treatment, n (%)		5 (63)
Employed, n (%)		6 (75)
**Highest level of education, n (%)**		
	No formal education	1 (13)
	Elementary education	0 (0)
	Lower secondary vocational education	1 (13)
	Secondary vocational education	1 (13)
	Upper secondary vocational education	0 (0)
	Higher vocational education	4 (50)
	Preuniversity education	0 (0)
	University education	0 (0)
	Postgraduate education	0 (0)
	Other	1 (13)

**Table 4:** Self-efficacy levels and phase of behavior change of participants for the physical activity and nutrition domains.

			Physical activity (n=7)		Nutrition (n=7)
Self-efficacy level, mean (SD)			29 (7)		32 (4)
**Phase of behavior change, n (%)**					
	Initiation	3 (43)		2 (29)	
	Action	1 (14)		0 (0)	
	Maintenance	3 (43)		5 (71)	

### Acceptability

#### Overview

**Figure 2:**
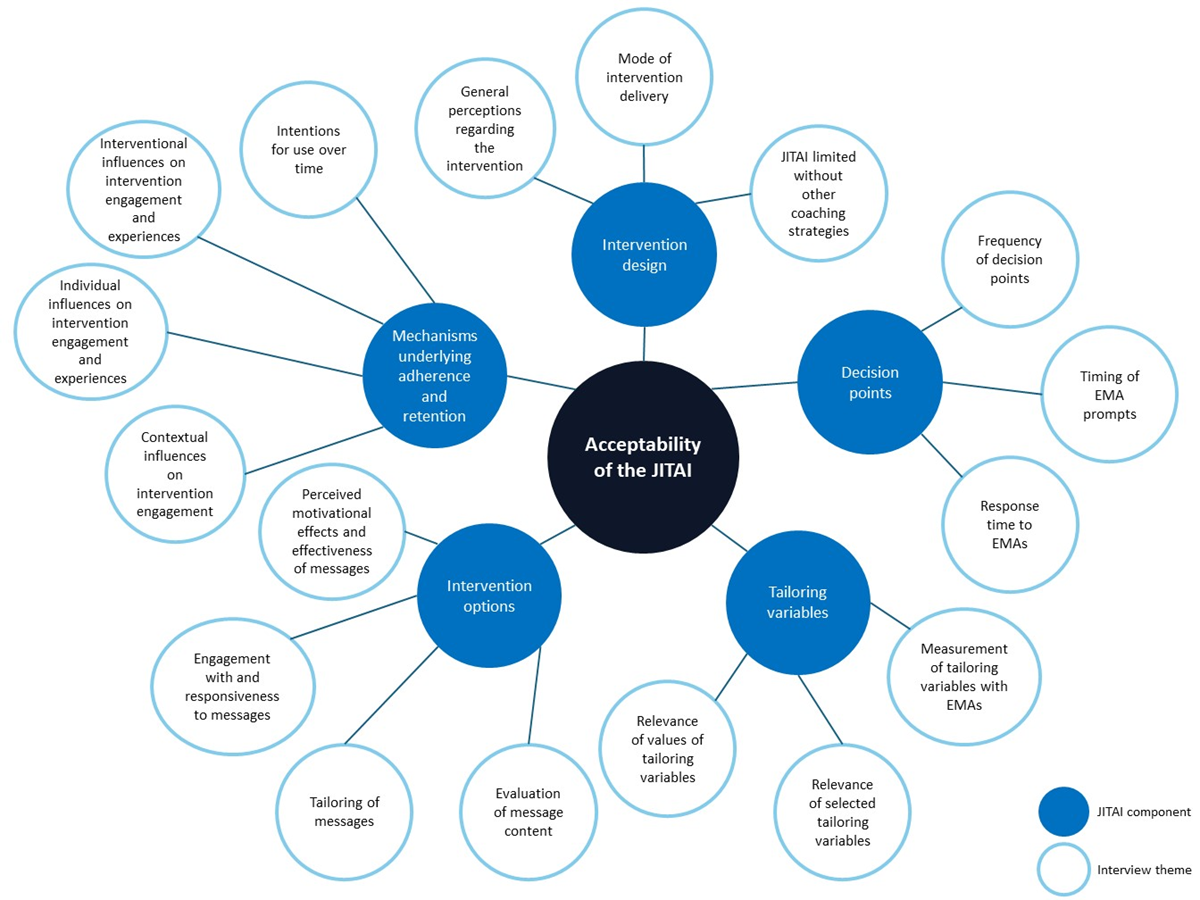
Overview of the just-in-time adaptive intervention (JITAI) components and associated themes identified from the interviews with individuals with type 2 diabetes. EMA: ecological momentary assessment.

Figure 2 [24] provides an overview of various JITAI components from the conceptual model of JITAIs by Nahum-Shani et al [24], with the themes identified from the interviews.

Table 5 presents the results of the interviews. The bold terms represent the various components from the conceptual model of JITAIs by Nahum-Shani et al [24], including the identified themes and their associated definitions that were identified from the interviews.

**Table 5:** Summary of the interview themes classified under JITAI^a^ components related to the acceptability of the JITAI.

JITAI components and identified themes			Definitions
**Intervention design**			
	General perceptions regarding the intervention	General perceptions about the intervention and associated components.	
	Mode of intervention delivery	Preferences regarding the method through which the intervention is delivered.	
	JITAI limited without other coaching strategies	Views on the need for additional support and the integration of other behavior change strategies.	
**Decision points**			
	Frequency of decision points	Perceptions and preferences regarding the frequency of EMA^b^ prompts and intervention options.	
	Timing of EMA prompts	Perceptions and experiences regarding the appropriateness and predictability of the timing of EMA prompts.	
	Response time to EMAs	Behaviors and perceptions regarding the timeliness of their responses to EMA prompts.	
**Tailoring variables**			
	Measurement of tailoring variables with EMAs	Perspectives on the usefulness and constraints associated with using EMAs to measure tailoring variables.	
	Relevance of selected tailoring variables	Perceptions of the appropriateness and usefulness of the tailoring variables included in the EMAs.	
	Relevance of values of tailoring variables	Perceptions of the suitability and adequacy of the response options provided for the tailoring variables in the EMAs.	
**Intervention options**			
	Evaluation of message content	Perceptions and assessments of the content of the messages.	
	Tailoring of messages	Perceptions regarding the personalization and relevance of the messages they received.	
	Engagement with and responsiveness to messages	Interactions with and reactions to the messages.	
	Perceived motivational effects and effectiveness of messages	Perceptions of how the messages participants received influenced their motivation and behavior change efforts.	
**Mechanisms underlying adherence and retention**			
	Contextual influences on intervention engagement	External factors that impact participants’ engagement with the intervention.	
	Individual influences on intervention engagement and experiences	Personal factors that affect participants’ engagement and experiences with the intervention.	
	Interventional influences on intervention engagement and experiences	Various factors related to the intervention itself that influence participants’ engagement and experiences.	
	Intentions for use over time	Intentions and willingness to continue using the JITAI system over a certain duration.	

^a^JITAI: just-in-time adaptive intervention.

^b^EMA: ecological momentary assessment.

#### Intervention Design

All intervention components received *average* to *good* ratings, ranging from 7 to 9, with all components scoring approximately equally.

Overall, participants found this JITAI to be an easy-to-use and generally useful concept for supporting a healthy lifestyle. In addition to providing advice on a healthy lifestyle, participants felt that it also made them more aware of their lifestyle choices for the day.

In addition, most participants appreciated receiving technology-supported lifestyle guidance. In this study, SMS text messaging was used, which was perceived as an easy medium. Most participants preferred using an app, although 1 participant found using an app more complicated than SMS text messaging. Participant 8 had a clear preference for using an app; he stated the following:

I do everything via my phone. I find using a phone or tablet excellent. ... I would actually enjoy that [using an app], it is the easiest thing there is. I do everything on my mobile. It is convenient if personal goals and medical records are also available on it.

Male, aged 59 years

However, some participants preferred in-person support or guidance, such as support from a spouse as social support or professional guidance from a health care provider. The preference for in-person guidance mainly stemmed from the ability to provide more context for certain events or feelings. Participant 3 illustrated this as follows:

For example, negative [mood] I will never indicate [in an EMA]. If I disagree with something, I will never put it in writing like that. If I have you on the phone personally, I will say what I feel, but on paper, that is not how I am. If I have to check on paper whether you are positive or negative, I will always indicate positive because if I indicate negative, I cannot provide any context, and then it is just how you want to read it and how you want to hear it. If I can provide an explanation to you, then I will more likely indicate that something is negative.

Male, aged 77 years

Some participants felt that relying solely on EMA-based messages to promote lifestyle was limited. They believed that coaching in this form alone lacked the pressure to actually change behavior. Examples to address this lack of pressure included setting goals and self-monitoring. Participant 1 had previously worked with goal setting and mentioned the following:

For example, the goal was: achieve 10,000 steps this week. And if I am missing 100, then I think: I will take a few more steps, I still want to reach those 10,000. It is an extra movement and pressure to do the exercise that I would not normally do, just to meet the set goal.

Male, aged 78 years

Other options mentioned were to make coaching more appealing by linking coaching to medical information about the participant or by providing healthy eating advice based on offers at the local supermarket.

#### Decision Points

For most participants, the number of prompts per day was acceptable, partly due to the low burden of completing the EMAs. However, some participants preferred a different prompt frequency, with options for both more and fewer EMA prompts mentioned. One participant suggested increasing the frequency from 3 to 4 EMA prompts per day, as it triggered engagement with a healthy lifestyle. Another participant preferred receiving the EMA prompts every other day, given their retired lifestyle with fewer varied activities.

The timing of the EMA prompts and messages was considered suboptimal by most participants. While they indicated that the timing was not disruptive, some prompts were deemed inconvenient, particularly when they occurred at times when participants could not respond, such as during work. Furthermore, some participants found the timing of the prompts predictable, as they often received them at approximately the same times each day, suggesting that more variation in timing would be preferred. Participant 5 elaborated on this, as follows:

For example, feeling hungry, that was often in the morning. But I had just eaten breakfast, so then I am not hungry and not craving anything. If you were to receive the questionnaire more in the evenings, that might be different, because then you’re more likely watching TV and, well, then you might have a different answer.

Female, aged 77 years

The occasionally unsuitable timing of the prompts and the fact that not everyone consistently had their mobile phone with them affected the time frame used to complete the EMAs. Some participants deliberately made time to respond to the prompts upon receiving them, while others waited for a suitable moment. None of the participants intentionally delayed completing the EMAs.

#### Tailoring Variables

One participant misunderstood the purpose of the EMA and did not grasp why they had to fill out a questionnaire so frequently, but overall, participants were satisfied with using an EMA to gain insight into their situation. However, two main points of criticism were that (1) EMA responses provide only a snapshot and (2) there was insufficient opportunity to provide context or additional explanations for the response options in the EMA.

Regarding the first point, participants noted that while an EMA is a useful tool to assess the situation at a specific moment, it lacks insight into what happens for the rest of the day and therefore does not adequately account for rapid changes that may occur in one’s situation, as explained by participant 3:

For example, I can be very negative in the morning, but after noon, I can be in a very good mood. I can bump my head and start swearing, and that can change at any moment. Therefore, it is a bit of a snapshot. That is why I often give standard answers, and I do not think you will gain much insight from that.

Male, aged 77 years

With regard to the second point, some participants felt there was insufficient opportunity to provide explanations for multiple-choice question answers. They would prefer an open field where they could freely elaborate on the multiple-choice questions.

In addition, participants felt a slight obligation to complete the EMAs because they felt researchers were waiting for their responses. Furthermore, some participants mentioned that they could not always answer completely freely due to feeling the need to provide socially desirable responses and being reluctant to give *negative* answers. However, all participants were comfortable sharing personal information, such as location or activity, via an EMA. The reasons for this included generally having no objection to sharing personal information, having given informed consent for this, and feeling a sense of autonomy by only providing information if they wished to do so.

Regarding the relevance of the tailoring variables, participants felt that the chosen variables were generally suitable for providing insights into their personal and environmental situations. Some participants mentioned that there could be room to include additional questions in the EMA without substantially increasing the burden. One participant suggested adding a specific question about experiencing hyperglycemia and hypoglycemia, so that this could also be taken into account in lifestyle advice. Furthermore, in some cases, certain questions were not relevant to a person, such as the mood question, which some participants mentioned always being in a good mood.

Most participants found the values of the tailoring variables, meaning the answer options in the EMA, sufficiently complete and clear. However, some improvement points were mentioned, such as making more distinctions in the answers for certain tailoring variables, for example, by including more types of weather conditions or also adding a neutral option in the *weather* tailoring variable. In addition, participant 5 indicated that the activity *eating* could not provide the researchers with enough information on the current activity. She made the following suggestion:

You could also fill in “eating,” I have done that before. Those things, it is clear, but it could be a bit clearer... For example, what am I eating? If you are eating your regular meal or if you are eating a snack. It would be nice if you can provide more information about that.

Female, aged 77 years

#### Intervention Options

Most participants found the content of the messages enjoyable, funny, and varied, with clear tips for improving lifestyle. Although there was some variation in how enjoyable participants found the messages, the content was not perceived as intrusive. Most feedback was related to the content of the recipes referenced in the messages. For example, 1 participant mentioned that the recipes were quite complex, requiring many ingredients that are not commonly used, leading to items expiring. Furthermore, some minor suggestions were made to improve the content of the messages, such as adding reminders.

Participants were generally satisfied with the degree of tailoring in the messages and could see that their EMA responses were reflected in the messages. However, participants also felt that there was variation in how well the messages aligned with individual circumstances. Participants noticed tailoring the most when they were addressed by their first names, when messages matched their personal preferences, and when messages matched their location or activity at that time. Participant 6 illustrated this with the following example:

Yes, I noticed from the little tips you got, for example asking a colleague for a walk during the break or traveling by bike to work instead of by car when you are at work.

Female, aged 55 years

Some participants had a more neutral opinion on the tailoring of the messages, such as that the messages sometimes still contained too much general knowledge or did not sufficiently align with their specific situation. Participant 4 believed that his preferences were considered, but he found that the messages did not sufficiently address his physical limitations:

Here it says at one point that it is good to move. I completely agree with you, but for me, it is currently difficult to move.... We used to do a lot of cycling and walking. I have had trouble with my right leg for five years now.

Male, aged 71 years

Participants indicated that they always read the messages, and some of them were even curious about the message they would receive based on the EMA, as indicated by participant 6:

It was fun. I was always waiting for the response I got. When I was at work, I got a message like: ask your colleague or do something with your colleague. When I had been to the store, I got different answers again, that was fun.

Female, aged 55 years

It was not always possible to read the messages immediately, but participants did so as soon as they could. Participants also showed a preference for interactivity, as several participants responded to the messages via SMS text messaging and expressed enjoyment in giving a reaction to the messages.

The responses regarding the motivating effect and perceived effectiveness of the messages varied. Almost all participants found receiving messages motivating to work on their lifestyle. Some participants indicated that they did not always act on the advice, partly because the advice was not always applicable to them. Nevertheless, it helped some participants make healthier choices, as participant 8 indicated with the following examples:

It did trigger me. Sometimes in the evening I thought: let’s get some fries. Then I thought: no, let’s just peel two potatoes, with a piece of meat and vegetables. I did that twice.

Male, aged 59 years

For example, that church service. Are you going to church? How? In that questionnaire, you indicate that you are going to do an activity. Then you get a message with: how could you improve this activity? Do you think about this? Are you going by bike, car, or walking? I started cycling, otherwise I would have taken the car.

Male, aged 59 years

#### Mechanisms Underlying Adherence and Retention

A contextual factor influencing engagement with the intervention was the COVID-19 pandemic and the lockdown period in the Netherlands during which the study was conducted. For instance, 1 participant mentioned that daily activities had substantially reduced due to the COVID-19 pandemic, referring to limited opening hours of stores and restaurants during the lockdown. As a result, the participant reported providing nearly identical answers every day, which lowered his engagement with the intervention. Another participant mentioned that they had become less active during the COVID-19 pandemic due to disruptions in their regular routine.

In addition, participants indicated various individual factors that influenced the use of the intervention. Some participants reported making many lifestyle adjustments or losing weight before participating in the study, making the intervention information less useful for them. Other participants expressed that they felt no need to improve their lifestyle because they were satisfied with their current health and lifestyle. Finally, some participants felt a slight resistance to actually adjusting their lifestyle, which seemed to be independent from the indicated phase of behavior change (Table 4). This feeling partly stemmed from their unwillingness to make certain lifestyle changes (eg, a participant who did not want to take the stairs) and from a desire to also be allowed to make unhealthy choices. Participant 2 already made many improvements to his lifestyle in recent years but indicated that he sometimes wanted to make unhealthy choices:

I am very careful with my eating. In the past 2.5 years, I have lost 18-20 kilos. I try to maintain my current weight now. That is certainly better for my diabetes too. But I do still snack occasionally.

Male, aged 78 years

The intervention itself also influenced engagement and experiences with the intervention. An important factor was the intervention burden. All participants found the burden of completing the EMA minimal. Participants found the EMA instructions and questions clear and the questionnaire brief, and it took little time to complete the EMA. They also found the link to the EMA questionnaire in the text message easy to use. As a result, participants remained motivated. However, the lack of variation in the EMA questionnaire reduced engagement over time for some participants. Because participants received the exact same questions every day, they found it monotonous. Several participants mentioned that they started filling out the questions on *autopilot*, as participant 5 described:

Overall, there was enough motivation, but because it was always the same questionnaire, you start thinking about it less. Only for activities like drinking coffee, I sometimes gave a different answer, but otherwise, it was always the same answer. You do not have to think about it anymore.

Female, aged 77 years

Participants suggested rearranging the questions or asking different questions on different days to make the EMA more engaging and encourage careful consideration when completing it.

In addition, participants provided feedback on the continued use of the intervention, with most participants willing to use the intervention for >2 weeks. Some participants were willing to fill out EMAs and receive messages daily without indicating a maximum period of use, but others saw more value in a maximum period of several weeks to months.

## Discussion

### Principal Findings

With this study, we aimed to gain insight into the acceptability of our initial JITAI version among individuals with T2D. This preliminary phase is pivotal for gaining insights into the target group’s experiences with the intervention, allowing us to assess and enhance its potential value based on their feedback [18]. Overall, participants expressed satisfaction with the intervention, as supported by their average to high ratings across its components. This opinion was evident from the positive feedback on the motivating and varied text messages, as well as some examples of personalized support that participants experienced.

It became evident that experiences varied substantially and were often influenced by individual and contextual factors that were not sufficiently captured with the EMAs. This variability was observed across all aspects of the intervention design. On the basis of the participants’ feedback, we have identified three key areas for improvement: (1) optimizing tailoring, (2) refining assessment methods, and (3) emphasizing integration with other behavioral intervention strategies.

Regarding the optimization of tailoring, our study highlighted the need for further refinement in this area. Although tailoring variables were selected based on target group input, their relevance varied among participants. For instance, mood- or disease-related symptoms were relevant to some but not all individuals. The decision to offer 2 prompts per day was informed by previous E-Supporter studies [35,36], yet participant feedback on prompt frequency varied. Similarly, we noted nonoptimal timing of prompts, aligning with challenges observed in related studies [57]. This heterogeneity among individuals’ experiences requires a shift toward individually tailored intervention designs. Realizing such individual tailoring can be approached in several ways. A straightforward method is to allow individuals to specify their preferences for tailoring variables, intervention options, as well as the frequency and timing of prompts before or during the intervention period [58,59]. Applying machine learning, such as reinforcement learning (RL), is a more advanced method for improving tailoring. RL learns and adapts from interaction and feedback. RL allows to tailor support strategies to individual needs and preferences regarding timing, frequency, and intervention types [60-63].

With respect to the assessment methods, participants mentioned the potential of EMAs to personalize intervention support, but they also identified limitations. These limitations included the repetitive nature of the EMA questionnaire and its snapshot insight into the current situation. Some participants reported reduced engagement over time, possibly attributed to the lack of variation in questions or the impact of the COVID-19 pandemic and associated restrictions during the study period [64]. Lockdown measures reduced social interactions and outdoor activities, leading to participants spending more time at home and engaging less in social or work-related activities. Consequently, responses to EMAs became predictable, and the participant’s interest diminished over time. EMAs rely on user input to deliver personalized support [27]. Decreased user engagement may lead to either skipping questions or responding without due consideration. This may influence the reliability and validity of measurements. As Nahum-Shani et al [24] emphasized, balancing response burden with the need for frequent assessment for capturing dynamic changes in certain variables is critical. Exploring variability in measured constructs across EMAs and considering alternative measurement methods for tailoring variables, such as GPS tracking for location and weather application programming interface, could simplify the data collection [57,61,65,66]. Nevertheless, issues such as privacy concerns with GPS and individuals’ perceptions of weather suitability for outdoor activities require consideration. While optimal measurement strategies for tailoring variables remain uncertain, we aim to find the right balance between passive and active methods for accommodating their variability in future interventions.

To realize sustainable behavior change, our study revealed that many participants found the intervention motivating, but some expressed no need, unwillingness, or resistance to follow the advice. The participants had T2D for an average of 15.6 (SD 7.7) years, which suggests they were already familiar with making lifestyle changes. This is supported by their relatively high self-efficacy scores, as individuals with high self-efficacy tend to feel confident in their ability to make behavioral changes. Consequently, their perceived need for external support from the intervention may have been reduced. Furthermore, many participants were already in the maintenance phase of behavior change, suggesting that they had already implemented some lifestyle changes. These individuals might require specific support for relapse prevention or, in some cases, no additional support at all [43]. A smaller group was in the initiation phase, indicating that they were just beginning to consider lifestyle changes. This group might require a stronger focus on motivational strategies to build commitment [1,2]. These differences highlight the need for interventions that can adapt to the wide variety of individual needs. However, JITAIs are generally designed to offer brief, just-in-time suggestions for immediate behavior adjustments [26,28,57]. While the JITAI may be effective for action-related changes, this design may fall short of addressing the more comprehensive strategies required. Therefore, we emphasize the importance of situating JITAIs within broader behavior change interventions. Integrating a JITAI within a comprehensive lifestyle intervention that includes extensive behavioral change strategies, as envisioned in our integration with the E-Supporter content, could provide the necessary support to facilitate sustainable behavior change.

### Strengths and Limitations

A strength of this study was the application of a hybrid approach to thematic analysis for analyzing the interviews. An advantage of this approach is that we were able to combine the strengths of both inductive and deductive approaches. The hybrid approach allowed us to use the flexibility of inductive coding to create data-driven insights and also use the deductive method’s ability to build upon existing knowledge, ensuring that the findings were both data driven and theoretically grounded.

A limitation of our study was the inability to measure the impact on proximal outcomes. Ideally, we would have supported the intervention with wearable activity monitoring and an app for dietary monitoring. However, due to COVID-19 restrictions preventing physical contact with participants, we were unable to distribute wearables or assist with app installations. In the future, our aim is to incorporate goal setting and self-monitoring into the intervention, as done in the E-Supporter. This approach can provide additional support in the behavior change process and enable examination of the intervention effects on proximal outcomes. As it was not the aim of this study to measure these outcomes, it does not affect the answer to the research question. Another limitation is the limited evaluation of intervention options for certain tailoring variables, such as negative mood or poor weather. As reported by participants, negative mood was seldom indicated in the EMA, resulting in no corresponding intervention options being sent. Furthermore, the evaluation was conducted during sunny weather, leading to minimal use of intervention options for poor weather conditions. Consequently, we lacked insight into the acceptability of these intervention options. However, the content and BCTs used in these intervention options are similar to others, so we anticipate similar perceptions of these messages compared to others. Finally, our sample size was relatively small, which may affect the robustness of the results. While this study provided valuable insights into the acceptability of the JITAI, our study population was relatively homogeneous, consisting predominantly of older individuals with a longer duration of diabetes. These characteristics appear to be a relatively good representation of the target group, characterized by an older average age, a slightly higher proportion of men than women, and including individuals with lower education levels, which are all sociodemographic factors associated with T2D [67,68]. However, the homogeneity limits the strength of our conclusions. Future research should focus on recruiting a more diverse population, including younger individuals and those with a shorter diabetes duration, to improve the generalizability of our findings.

### Conclusions

This study presents the first version of an EMA-driven JITAI designed to support a healthy lifestyle in individuals with T2D. The intervention received positive feedback, particularly for its motivating messages that felt tailored. However, individual differences in experiences highlight the need for improvement in tailoring, assessment methods, and the integration of JITAI with other behavioral intervention strategies.

## Appendix

Multimedia Appendix 1 E-Supporter determinants and behavior change techniques.

Multimedia Appendix 2 Translated interview schedule.

## Abbreviations

BCT: behavior change technique
EMA: ecological momentary assessment
ESES: Exercise Self-Efficacy Scale
JITAI: just-in-time adaptive intervention
RL: reinforcement learning
T2D: type 2 diabetes
ZGT: Ziekenhuisgroep Twente

## Data Availability

The data generated during this study are not publicly available because of the personal information shared during the interviews but are available from the corresponding author on reasonable request.
