# Supplementary material for: Exploring the Acceptance of Just-in-Time Adaptive Lifestyle Support for People With Type 2 Diabetes: Qualitative Acceptability Study
Source: JMIR Form Res. 2025 Feb 19;9:e65026. doi: 10.2196/65026 (PMC11888104; doi:10.2196/65026)
Supplement: Multimedia Appendix 1 [file formative_v9i1e65026_app1.pdf]

Multimedia Appendix 1. E-Supporter Determinants and Behavior Change Techniques (BCTs)

Table S1. Determinants and linked BCTs in the JITAI

	Action control	Self-efficacy	Knowledge	Risk perception	Outcome expectations	Attitude	Social support	Action planning	Coping planning	Behavioral cueing [a]	Environmental context [a]	Mood management	Habits	Satisfaction	Social influences
1.1 Goal setting (behavior)		V													
1.2 Problem solving		V						V	V				V		
1.4 Action planning		V						V	V	V			V		
1.5 Review behavior goal	V														
2.2 Feedback on behavior	V														
2.3 Self-monitoring of behavior	V	V													
2.4 Self-monitoring of outcome(s) of behavior														V	
3.1 Social support, including motivational interviewing		V					V								V
3.2 Social support (practical)							V				V				V
3.3 Social support (emotional)							V								V
4.1 Instruction on how to perform the behavior		V	V												
5.1 Information about health consequences			V	V	V	V									
5.6 Information about emotional consequences					V										
6.3 Information about others' approval															V
7.1 Prompts/cues								V		V	V		V		
8.2 Behavior substitution [a]	V									V			V		
8.3 Habit formation													V		
8.4 Habit reversal [a]										V			V		
9.1 Credible source						V									
9.2 Pros and cons					V	V									
9.3 Comparative imagining of future outcomes					V										
11.2 Reduce negative emotions		V										V			
12.1 Restructuring the physical environment [a]										V	V				
12.2 Restructuring the social environment [a]											V				
12.3 Avoidance/reducing exposure to cues for the behavior [a]										V	V		V		
12.5 Adding objects to the										V	V				

environment [a]															
13.2 Framing/reframing						V									
15.1 Verbal persuasion about capability		V													
15.3 Focus on past success		V													

[a] Determinants of behavior or BCTs that were added in the JITAI but were not yet part of the E-Supporter 1.0
