# Supplementary material for: Exploring the Acceptance of Just-in-Time Adaptive Lifestyle Support for People With Type 2 Diabetes: Qualitative Acceptability Study
Source: JMIR Form Res. 2025 Feb 19;9:e65026. doi: 10.2196/65026 (PMC11888104; doi:10.2196/65026)
Supplement: Multimedia Appendix 2 [file formative_v9i1e65026_app2.pdf]

Multimedia Appendix 2. Translated interview schedule

1. Introduction

Good day,

This is *<name>, <function>*, speaking. I am calling because we have scheduled an interview for today. Over the past two weeks, you participated in our study, where you received daily short questionnaires and text messages with advice on leading a healthier lifestyle. Today, we would like to ask you some questions about your experience with the questionnaires and messages. Your opinion is what matters, so there are no right or wrong answers. If you do not understand a question, please let me know.

The interview consists of three parts, during which I would like to ask you about the following topics:

- Your opinion on the daily questionnaires (i.e., the EMAs)
- Your opinion on the daily text messages
- Your opinion on the overall experience with the questionnaires and messages

If you agree, I will record this interview so that I can review your responses later. Your name will not be used in the recording. As stated in the study information, these audio recordings will only be used for research purposes. Do you still agree with this? Do you have any questions before we start the interview? If you have no further questions, we can begin the interview.

<Start audio recording>

2. Interview questions

Table S2. Interview topics and questions

**Topic**	**Questions**
Experiences with the EMAs (daily questionnaire(s))	Every day, you received two short questionnaires asking about your situation at a particular time of day, such as what you were doing or the weather. The following questions are about these daily questionnaires.<br><br>The questionnaire began with a brief instruction on what was expected of you when filling it out.<br><br>• What did you think of these instructions for completing the questionnaire?<br>o Did these instructions provide the information you needed to complete the questionnaire?<br>*(If no: probe further to understand why not.)*<br>o What did you think about the amount of instruction provided?<br>*(If too much or too little instruction: probe further to find out what the participant would like to see changed.)*<br>o Would you have liked to receive any other instructions before starting the questionnaire?<br>*(If yes: probe further to understand what additional instructions they would have wanted.)*

<table>
<tr>
<td></td>
<td>
Following the instructions, the questionnaire asked questions about, among other things, your location, activity, and mood. This was intended to provide you with personalized advice for physical activity and nutrition that suited your situation at that time. The next questions are about the content of the questionnaire.

Were the questions in the questionnaire easy to understand?
(If not, probe further to identify which questions were unclear.)
What did you think of the questions designed to give insight into your situation at that moment?

Which questions did you find useful to give information about your situation?
(Probe further to understand why.)
Which questions did you find less useful to give information about your situation?
(Probe further to understand why.)
Did you feel any questions were missing that would help provide personalized advice on physical activity or nutrition?
(If yes, probe further to understand what the participant felt was missing.)

Each question also included various answer options, meaning the possible responses you could give. The next questions are about these answer options.

Were the answer options provided clear?
(If not, probe further to understand which answer options were unclear.)
What did you think of the provided answer options for the questions?

Were there any answer options that were less suitable?
(If yes, probe further to identify which answer options.)
Did you feel any answer options were missing that would give a better picture of your situation?
(If yes, probe further to understand what participants felt was missing.)

Next, I would like to ask a few questions about your perception of the burden of the questionnaires.

Could you describe what you thought about the burden of the questionnaires?

What did you think about the length of the questionnaires?
(If too long or short: probe further to understand why.)
What did you think about the number of questionnaires you received per day?
(If too many or too few: probe further to understand why.)

Did you feel motivated to fill out the questionnaires?
(Probe further to understand why.)

Did your motivation to fill out the questionnaires change over time?
(If yes, probe further to understand why.)

What did you think about us asking for personal information such as your location, activity, and mood?
(Consider: intrusiveness regarding privacy.)

</td>
</tr>
<tr>
<td>Experiences with the text messages</td>
<td>
In addition to the questionnaires, you received two text messages per day with advice for a healthy lifestyle. The following questions are about these text messages.

Can you tell me what you thought about the text messages?

What did you think of the advice provided in the text messages?
(Probe further to understand why participants held certain opinions.)

</td>
</tr>
</table>

	○ To what extent did the messages feel personalized? Consider factors such as your mood, activities, and the weather.<br>*(Probe further to understand why this was or was not perceived as personal.)*<br>▪ Can you give examples of messages that felt personal to you?<br>*(Probe further to understand why these were considered good messages.)*<br>▪ Can you give examples of messages that did not feel personal to you?<br>*(Probe further to understand why these were considered less suitable messages.)*<br>• Is there anything you would like to see changed in the text messages?<br>*(If yes, probe further to understand what changes the participant would prefer.)*
Experiences with the JITAI overall	In this final section, I would like to ask you some questions about receiving lifestyle advice in this way, focusing on the questionnaires and messages together.<br><br>• What did you generally think about this method of receiving lifestyle advice?<br>*(Probe further to understand why the participant held certain opinions.)*<br>• Do you think this advice has helped you to lead a healthier lifestyle, such as being more active or eating healthier?<br>*(Probe further to understand why participants believe this does or does not contribute to a healthy lifestyle.)*<br>○ Can you give examples of healthy choices you made because of the advice?<br>• What did you think about the ease of use of the questionnaires and messages?<br>*(If it was not easy to use, probe further to understand what was challenging about using the intervention.)*<br>• When you received a questionnaire or text message, did you complete or read it immediately?<br>*(If not, probe further to understand the reasons for not responding immediately.)*<br>• You have now used these questionnaires and messages for two weeks. Would you be willing to use these questionnaires and messages for a longer period?<br>*(Probe further to understand why the participant would or would not want to use the intervention longer.)*<br><br>Finally, I would like to ask you to rate the coaching module.<br>*(ask for each rating for a short explanation)*<br><br>• What rating would you give the questionnaires on a scale from 1 (very poor) to 10 (excellent)?<br>• And what rating would you give the text messages?<br>• And what rating would you give the entire module?

3. Conclusion

This is the end of this interview. Do you have any questions or experiences we have not discussed yet? We will use your input to further improve this concept of lifestyle coaching via mobile phone. I would like to thank you for your effort and time in participating in this interview.

<Stop recording>
